# Low Annexin A1 expression predicts benefit from induction chemotherapy in oral cancer patients with moderate or poor pathologic differentiation grade

**DOI:** 10.1186/1471-2407-13-301

**Published:** 2013-06-21

**Authors:** Dong-wang Zhu, Ying Liu, Xiao Yang, Cheng-zhe Yang, Jie Ma, Xi Yang, Jin-ke Qiao, Li-zhen Wang, Jiang Li, Chen-ping Zhang, Zhi-yuan Zhang, Lai-ping Zhong

**Affiliations:** 1 Department of Oral & Maxillofacial-Head & Neck Oncology, Ninth People’s Hospital, College of Stomatology, Shanghai Jiao Tong University School of Medicine, No.639 Zhizaoju Road, Shanghai 200011, China; 2 Department of Oral Pathology, Ninth People’s Hospital, College of Stomatology, Shanghai Jiao Tong University School of Medicine, No.639 Zhizaoju Road, Shanghai 200011, China

**Keywords:** Annexin A1, Oral squamous cell carcinoma, Induction chemotherapy

## Abstract

**Background:**

The benefit of induction chemotherapy in locally advanced oral squamous cell carcinoma (OSCC) remains to be clearly defined. Induction chemotherapy is likely to be effective for biologically distinct subgroups of patients and biomarker development might lead to identification of the patients whose tumors are to respond to a particular treatment. Annexin A1 may serve as a biomarker for responsiveness to induction chemotherapy. The aim of this study was to investigate Annexin A1 expression in pre-treatment biopsies from a cohort of OSCC patients treated with surgery and post-operative radiotherapy or docetaxel, cisplatin and 5-fluorouracil (TPF) induction chemotherapy followed by surgery and post-operative radiotherapy. Furthermore we sought to assess the utility of Annexin A1 as a prognostic or predictive biomarker.

**Methods:**

Immunohistochemical staining for Annexin A1 was performed in pre-treatment biopsies from 232 of 256 clinical stage III/IVA OSCC patients. Annexin A1 index was estimated as the proportion of tumor cells (low and high, <50% and ≥50% of stained cells, respectively) to Annexin A1 cellular membrane and cytoplasm staining.

**Results:**

There was a significant correlation between Annexin A1 expression and pathologic differentiation grade (P=0.015) in OSCC patients. The proportion of patients with low Annexin A1 expression was significantly higher amongst those with moderate/poorly differentiated tumor (78/167) compared to those with well differentiated tumor (18/65). Multivariate Cox model analysis showed clinical stage (P=0.001) and Annexin A1 expression (P=0.038) as independent prognostic risk factors. Furthermore, a low Annexin A1 expression level was predictive of longer disease-free survival (P=0.036, HR=0.620) and locoregional recurrence-free survival (P=0.031, HR=0.607) compared to high Annexin A1 expression. Patients with moderate/poorly differentiated tumor and low Annexin A1 expression benefited from TPF induction chemotherapy as measured by distant metastasis-free survival (P=0.048, HR=0.373) as well as overall survival (P=0.078, HR=0.410).

**Conclusions:**

Annexin A1 can be used as a prognostic biomarker for OSCC. Patients with moderate/poorly differentiated OSCC and low Annexin A1 expression can benefit from the addition of TPF induction chemotherapy to surgery and post-operative radiotherapy. Annexin A1 expression can potentially be used as a predictive biomarker to select OSCC patients with moderate/poorly differentiated tumor who may benefit from TPF induction chemotherapy.

## Background

Oral squamous cell carcinoma (OSCC) is the most common type of head and neck cancer. Patients with OSCC have poor clinical outcomes including treatment related organ dysfunction. The 5-year survival rate of OSCC patients is 50-60% [1,2]. To improve the clinical management of OSCC patients, it is important to develop different treatment strategies and ways to determine which subgroup of patients respond mostly to various strategies. Currently, the most common treatment for patients with locally advanced and resectable OSCC is radical surgery followed by post-operative radiotherapy or chemoradiotherapy depending on the presence of high-risk features in the surgical specimen. Clinically, only clinical staging and pathologic differentiation grade are used to predict prognosis of OSCC patients [3-5]. Therefore, it is critical to understand the biological basis of OSCC and develop novel biomarkers that can help predict the prognosis and likelihood that a patient benefits from a particular treatment strategy.

Induction chemotherapy is regarded as an effective way to reduce locally advanced or aggressive cancers, to improve the chance of eradication of locoregional lesions by radical surgery and/or radiotherapy/chemoradiotherapy, and to preserve end-organ functionality to ultimately maintain a high quality of life. Recently, two randomized phase 3 trials (TAX323 and TAX324) demonstrated that induction chemotherapy protocol of docetaxel, cisplatin and 5-fluorouracil (TPF) combination followed by radiotherapy or chemoradiotherapy can improve overall survival (OS) compared to cisplatin and 5-fluorouracil (PF) in head and neck squamous cell carcinoma (HNSCC) patients [6-8]. However, it is still unknown whether TPF induction chemotherapy improves outcomes when given prior to surgery in patients with locally advanced HNSCC, especially OSCC. To address the role of induction TPF in OSCC treated with surgery, we conducted a randomized phase 3 trial of induction TPF followed by surgical resection versus surgical resection in patients with locally advanced OSCC [9]. We failed to demonstrate a survival advantage for induction chemotherapy in the overall study population. It is possible, however, that induction chemotherapy with TPF might improve outcomes in a molecularly defined subgroup of patients. Correlative studies from the aforementioned randomized trials could assist in identifying candidate biomarkers predictive of benefit from induction treatment.

Annexin A1 is an intracellular protein that can bind calcium and phospholipids. It has been suggested to have an important role in the inflammation response, cell proliferation, cell signaling, phagocytosis, and carcinogenesis [10]. Although there is still controversy regarding Annexin A1 expression in different types of cancers, including breast, pancreatic, hepatic, prostate, urothelial, cervical, and head and neck cancer [11-27], low Annexin A1 expression correlates with poor pathologic differentiation grade [11-15]. In fact, absence of Annexin A1 expression has been reported to correlate with a poor pathologic response to induction chemotherapy in breast cancer [28]. However, the clinical usefulness of Annexin A1 expression in OSCC is not well understood. Specifically, it is unknown if Annexin A1 expression in the pre-treatment biopsy from OSCC patients can be used as a prognostic biomarker or an indication for induction chemotherapy.

The aim of the present study was to evaluate Annexin A1 expression in the pre-treatment biopsy specimens from patients with resectable locally advanced OSCC. These patients had been enrolled in a randomized phase 3 trial of TPF induction chemotherapy followed by surgery and post-operative radiotherapy compared to surgery and post-operative radiotherapy. Furthermore we aimed to examine the possible prognostic and predictive role of Annexin A1expression in this patient population. We hypothesize that low Annexin A1 expression plays a role in survival of patients with OSCC, and is also predictive of patient benefit from TPF induction chemotherapy.

## Methods

### Patients

256 patients with primary and locally advanced OSCC were enrolled in a prospective, randomized, phase 3 trial at Ninth People’s Hospital, Shanghai Jiao Tong University School of Medicine [9], which was in compliance with the Helsinki Declaration. The aim of this study was to test the hypothesis that TPF induction chemotherapy administered prior to surgery and post-operative radiotherapy improves survival in patients with resectable locally advanced OSCC (trial registration ID: NCT01542931). After eligibility was confirmed and written informed consent obtained, patients were randomized to the control group (surgery followed by post-operative radiotherapy) or experimental group (TPF induction chemotherapy followed by surgery and post-operative radiotherapy).

The TPF induction chemotherapy consisted of docetaxel 75 mg/m^2^ intravenously and cisplatin 75 mg/m^2^ intravenously on day 1, followed by 5-fluorouracil 750 mg/m^2^/day as a 120-hour continuous intravenous infusion on days 1 through 5. Induction chemotherapy was given every 3 weeks for 2 cycles. Surgery was performed at least 2 weeks after completion of induction chemotherapy, consisting of radical resection of the primary lesion and full neck dissection with appropriate reconstruction (pedicle or free flap); frozen sections during surgery was performed to confirm adequate margins. Post-operative radiotherapy was initiated 4–6 weeks after surgery, at a dose of 1.8-2 Gy/day, 5 days/week for 6 weeks, totally 54-60 Gy; in patients with high risk features, such as positive surgical margins, extracapsular nodal spread, or vascular embolism, a total radiation dose of 66 Gy was recommended.

Clinical tumor response to induction chemotherapy was determined by clinical evaluation and imaging studies (performed at baseline and 2 weeks after cycle 2 of induction chemotherapy). Responses were characterized according to the RECIST version 1.0 [29]. Pathologic response to TPF induction chemotherapy was assessed by examination of the resected specimen. A favorable response was defined as absence of tumor cells or presence of scattered foci of a few tumor cells (minimal residual disease with <10% viable tumor cells), as previously described by Licitra et al. [30]; an unfavorable pathologic response was defined as the presence of ≥10% viable tumor cells in the resected specimen.

After treatment, patients were monitored every three months in the first two years, every six months in the subsequent 3–5 years, and once a year thereafter until death or data censoring.

### Detection of Annexin A1 expression using immunohistochemistry

Pre-treatment formalin fixed and paraffin embedded biopsy specimens were used for detection of Annexin A1 expression; however, in the control group, if pre-treatment biopsy was unavailable, resected surgical specimens were used. Sections of 4 *μ*m thick were studied using hematoxylin and eosin (HE) staining and immunohistochemical staining for Annexin A1. The HE sections were reviewed according to the WHO histological criteria [31]. Immunohistochemical staining was accomplished using well established methods as previously described [32,33]. In brief, after deparaffinization, endogenous peroxidase block and heat-induced epitope retrieval, primary rabbit polyclonal antibody to Annexin A1 (product code of BA0640, Boster Biotech Co., Wuhan, China) at 1:150 dilution was added overnight at 4°C, then visualized using 3,3’-diaminobenzidine (DAB) detection kit (Dako Cytomation, Denmark). The 1:150 dilution was the best dilution compared to 1:50, 1:100, and 1:200. Negative control was prepared using PBS instead of antibody. Microscopic examination was performed by two pathologists and all specimens were blinded. Positive staining for Annexin A1 expression was observed in the cellular membrane and cytoplasm. The Annexin A1 expression index was determined based on the proportion of stained cells on a scale of negative to strong as follows: negative, absence of stained cells; weak positive, <50% of stained cells; and strong positive, ≥50% of stained cells. Low Annexin A1 expression was defined as negative and weak positive Annexin A1 expression, high Annexin A1 expression was defined as strong positive Annexin A1 expression. This was based on previous studies demonstrating that the chosen cut-off of 50% was reasonable for prognostic analysis [24].

### Statistical analysis

The primary endpoint of this trial was survival rate. Second endpoints of this trial were local control and safety. Overall survival (OS) was calculated from the date of randomization to the date of death; disease-free survival (DFS)/locoregional recurrence-free survival (LRFS)/distant metastasis-free survival (DMFS) were calculated, respectively, from the date of randomization to recurrence/locoregional recurrence/distant metastasis or death from any cause.

For descriptive analysis, categorical data were expressed as number and percentage. Chi-square test was applied to compare the difference between the baseline factors and Annexin A1 expression. The survival analysis was conducted using the Kaplan-Meier method and log-rank test. Hazard ratios (HR) were calculated using the Cox proportional hazards model. Intention-to-treat principle was applied for efficacy analysis.

All hypothesis-generating tests were two-sided at a significance level of 0.05. Data were analyzed with the statistical software SPSS13.0 for Windows (SPSS Inc., USA).

## Results

### Annexin A1 expression in OSCC patients

**Figure 1:**
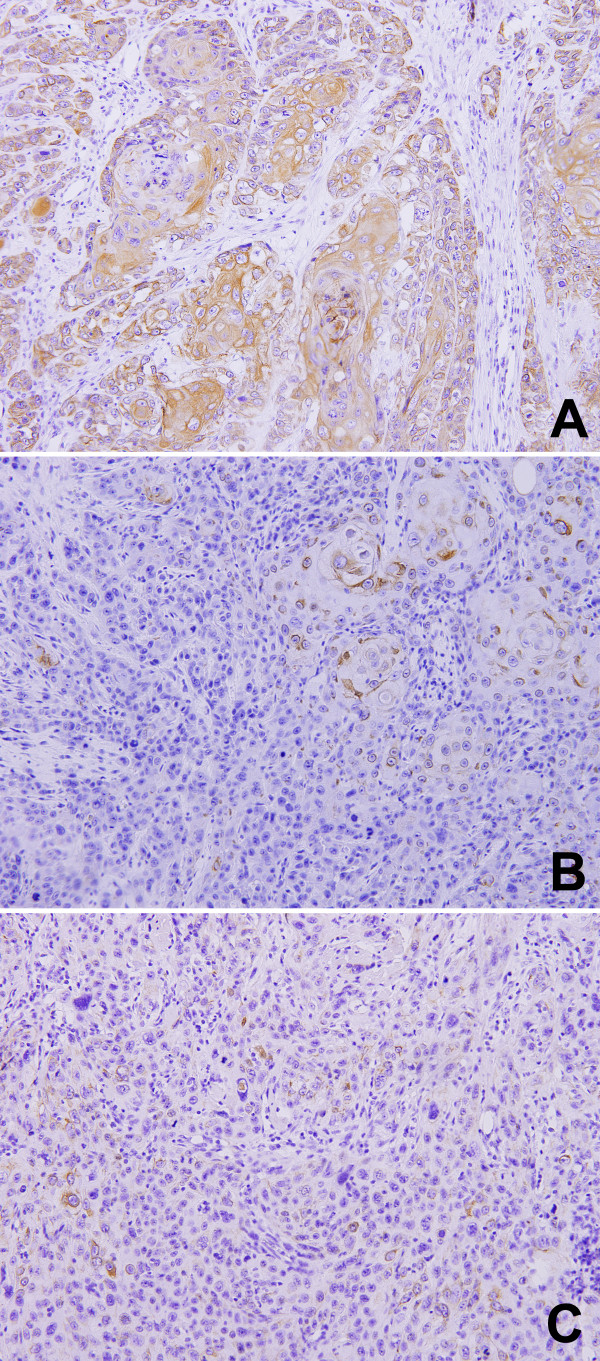
**Immunohistochemical staining for Annexin A1 in the pre-treatment biopsy samples from oral squamous cell carcinoma patients. (A)** Well differentiated grade, **(B)** Moderately differentiated grade, **(C)** Poorly differentiated grade.

From 03/2008 to 12/2010, 256 eligible patients were enrolled in this trial (128 patients in each group). 232 (91%, 127 patients in the control group, 105 patients in the experimental group) patients were assessed for pre-treatment tumor Annexin A1 expression levels. Table 1 summarizes their baseline clinical characteristics, with no significant imbalance between the two groups. 96 specimens (56 in the control group and 40 in the experimental group) demonstrated low Annexin A1 expression, and 136 specimens (71 in the control group and 65 in the experimental group) exhibited high Annexin A1 expression. There was an equal distribution of Annexin A1 expression between the two groups (Chi-square test=0.853, P=0.356). No significant difference of proportion of Annexin A1 expression was found according to baseline characteristics with exception of pathologic differentiation grade and alcohol use (Table 1). The proportion of patients with low Annexin A1 expression was higher amongst patients with moderate/poorly differentiated tumor (Figure 1) (78/167) and positive alcohol use (44/88) compared to those with well differentiated tumor (Figure 1) (18/65) and negative alcohol use (52/144), respectively There was no significant difference between pathologic differentiation grade and alcohol use (P=0.499).

**Table 1:** Baseline characteristics and Annexin A1 expression

**Characteristics**	**Total**	**Annexin A1 expression**		P value*
	**patients**	**Low**	**High**	
	**N=256**	**N=96**	**N=136**	
	**n (%)**	**n (%)**	**n (%)**	
Gender				
Male	179 (69.9)	72 (77.2)	88 (61.0)	0.095
Female	77 (30.1)	24 (22.8)	48 (39.0)	
Age (years)				
<60	168 (65.6)	67 (71.1)	90 (64.4)	0.562
≥60	88 (34.4)	29 (29.9)	46 (35.6)	
Site				
Tongue	113 (44.1)	42 (42.1)	56 (42.4)	0.281
Buccal	45 (17.6)	15 (17.5)	28 (20.3)	
Gingiva	40 (15.6)	11 (12.3)	27 (21.1)	
Floor of mouth	30 (11.7)	16 (16.7)	13 (8.5)	
Palate	18 (7.0)	7 (6.1)	7 (5.9)	
Retromolar trigone	10 (3.9)	5 (6.1)	5 (2.5)	
Clinical T descriptor				
T1/T2	66 (25.8)	23 (24.6)	38 (28.0)	0.497
T3/T4	190 (74.2)	73 (75.4)	98 (72.0)	
Clinical N descriptor				
N0	110 (43.0)	40 (39.5)	59 (45.8)	0.480
N1	94 (36.7)	33 (39.5)	53 (34.7)	
N2	52 (20.3)	23 (21.0)	24 (19.5)	
Clinical stage				
III	177 (69.1)	60 (65.8)	100 (72.0)	0.074
IVA	79 (30.9)	36 (34.2)	36 (28.0)	
Pathologic differentiation				
Well	80 (31.2)	18 (22.8)	47 (33.1)	0.015
Moderately	165 (64.5)	71 (71.1)	85 (63.6)	
Poorly	11 (4.3)	7 (6.1)	4 (3.4)	
Smoking status**				
Current/former	126 (49.2)	52 (56.1)	58 (39.0)	0.084
Never	130 (50.8)	44 (43.9)	78 (61.0)	
Alcohol use***				
Positive	98 (40.6)	44 (48.2)	44 (28.0)	0.037
Negative	158 (59.4)	52 (51.8)	92 (72.0)	

*P value from the chi-square test was reported to compare the difference between low and high Annexin A1 expression based on the different baseline factors.

**Former/current smokers defined as at least a one pack-year history of smoking.

***Positive alcohol use was defined as current alcohol use of more than one drink per day for 1 year (12 ounces of beer with 5% alcohol, or 5 ounces of wine with 12%-15% alcohol, or one ounce of liquor with 45%-60% alcohol). All other patients were classified as negative alcohol use.

### Annexin A1 expression and response to induction chemotherapy

In the experimental group, responses by RECIST in 105 patients with assessment of Annexin A1 that initiated induction chemotherapy were: 78.1% clinical response (4 patients with complete response and 78 patients with partial response) and 18.1% clinical non-response (18 patients with stable disease and 1 patient with progressive disease), 4 patients were unevaluable for response. Favorable and unfavorable pathologic responses were observed in 26.7% (27/101) and 73.3% (74/101) of patients, respectively. Pathologic response could not be evaluated in 4 patients. Annexin A1 expression did not correlate with clinical response to TPF induction chemotherapy (Chi-square test=1.073, P=0.300), or the pathologic response to induction chemotherapy (Chi-square test=1.820, P=0.177) (Table 2), even when stratified according to alcohol use (Cochran’s Mantel-Haenszel test=0.313, P=0.576 for clinical response; Cochran’s Mantel-Haenszel test=0.488, P=0.485 for pathologic response).

**Table 2:** Clinical and pathologic response to TPF induction chemotherapy according to Annexin A1 expression

	**Annexin A1 expression**		**Chi-square**
	**Low**	**High**	**test P value**
Clinical response	32	50	0.300
Clinical non-response	5	14	
Favorable pathologic response	7	20	0.177
Unfavorable pathologic response	30	44	

### Annexin A1 expression and patients’ outcomes

**Figure 2:**
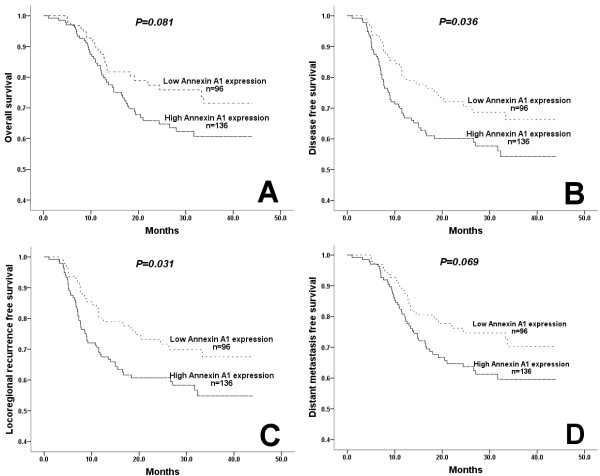
**Overall survival, disease-free survival, locoregional recurrence-free survival, distant metastasis-free survival in the patients with low and high Annexin A1 expression.** A trend of low Annexin A1 expression indicating a better overall survival **(A)** and distant metastasis-free survival **(D)** compared to high Annexin A1 expression; however, a low Annexin A1 expression significantly indicating a better disease-free survival **(B)** and locoregional recurrence-free survival **(C)** compared to high Annexin A1 expression.

No patients were lost to follow-up; the median follow-up time was 30 months among the censored patients. There was no significant difference on OS, DFS, LRFS or DMFS between the patients with and without TPF induction chemotherapy. The estimated 2-year OS was 68.2% and 68.8% in the patients with and without TPF induction chemotherapy, respectively. Locoregional recurrence and distant metastasis occurred in 30.9% and 7.0%, respectively. In general, no significant difference was seen in locoregional recurrence or distant metastasis rates between the patients with and without TPF induction chemotherapy. However, in the experimental group, the patients with low Annexin A1 expression had a significantly lower local recurrence rate compared to that in the control group (Table 3). Survival analysis showed that the patients with low Annexin A1 expression had a better survival, especially the DFS (P=0.036, HR=0.620) and LRFS (P=0.031, HR=0.607) (Figure 2). Univariate Cox model was used to analyze the impact of baseline characteristics on the time-to-event endpoints; Annexin A1 expression (low vs. high), lymph node status (cN0-1 vs. cN2, or cN0 vs. cN1-2), and clinical stage (stage III vs. stage IVA) were risk factors on OS, DFS, LRFS or DMFS. Multivariate Cox model analysis was performed using the risk factors of Annexin A1 expression and clinical stage; while lymph node status (cN0-1 vs. cN2 or cN0 vs. cN1-2) was not used because of the direct correlation between clinical stage and lymph node status. Both the clinical stage (P=0.001) and Annexin A1 expression (P=0.038) were independent risk factors. When pathologic differentiation grade and alcohol use were used in the multivariate Cox model analysis, only the clinical stage (P=0.001) and Annexin A1 expression (P=0.048) were independent risk factors.

**Table 3:** Comparison of local/regional/distant failure between low and high Annexin A1 expression in the oral squamous cell carcinoma patients treated with or without TPF induction chemotherapy

**Characteristics**		**Annexin A1 expression**		**Chi-square test**	**Cochran Mantel Haenszel test**
		**Low**	**High**	**P value**	**P value**
Surgery+post-operative radiotherapy					0.020
	No local failure	45	52	0.348	
	Local failure	11	19		
TPF+surgery+post-operative radiotherapy					
	No local failure	37	47	0.012	
	Local failure	3	18		
Surgery+post-operative radiotherapy					0.332
	No regional failure	51	60	0.268	
	Regional failure	5	11		
TPF+surgery+post-operative radiotherapy					
	No regional failure	34	54	0.795	
	Regional failure	6	11		
Surgery+post-operative radiotherapy					0.367
	No distant failure	52	65	0.786	
	Distant failure	4	6		
TPF+surgery+post-operative radiotherapy					
	No distant failure	40	62	0.168	
	Distant failure	0	3		

### Annexin A1 expression, pathologic differentiation grade and patients’ outcomes

Patients with well differentiated tumor had a better outcome than those with moderate/poorly differentiated tumor. Pathologic differentiation grade did not have a significant effect on outcomes of the entire cohort with respect to OS (P=0.250), DFS (P=0.679), LRFS (P=0.790), and DMFS (P=0.260). In the experimental group, patients with well differentiated tumor had a better OS (P=0.958), DFS (P=0.711), LRFS (P=0.711), and DMFS (P=0.972) than those with moderate/poorly differentiated tumor. This was similar to the control group with respect to OS (P=0.132), DFS (P=0.415), LRFS (P=0.524), and DMFS (P=0.162).

**Figure 3:**
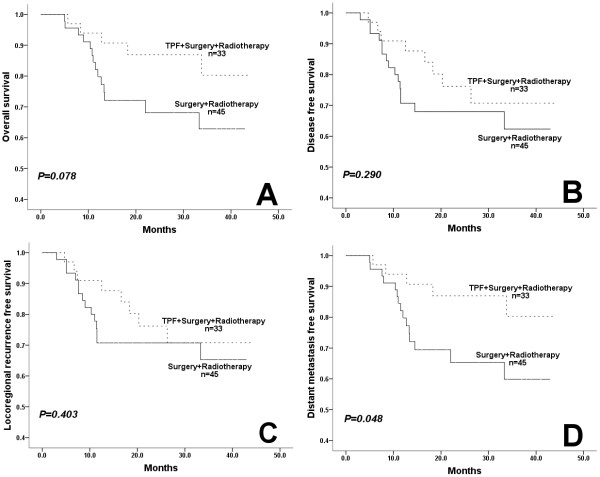
In patients with moderate/poorly differentiated tumor, those with low Annexin A1 expression benefited from TPF induction chemotherapy on overall survival (A) and distant metastasis-free survival (D), but not benefit from TPF induction chemotherapy on disease-free survival (B) or locoregional recurrence-free survival (C).

In patients with well differentiated tumor, there was no significant difference in OS, DFS, LRFS or DMFS between patients treated with or without TPF induction chemotherapy, regardless of Annexin A1 expression. In patients with moderate/poorly differentiated tumor, low Annexin A1 expression benefited from TPF induction chemotherapy in OS (P=0.078, HR=0.410) and DMFS (P=0.048, HR=0.373) (Figure 3); however, patients with high Annexin A1 expression did not benefit from TPF induction chemotherapy.

## Discussion

In this study, we found that Annexin A1 may be used as a prognostic biomarker in locally advanced and resectable OSCC patients. Specifically, a lower Annexin A1 expression indicates improved survival. Annexin A1 expression correlates with pathologic differentiation grade of biopsy specimens from OSCC patients. A lower Annexin A1 expression correlates with a poorer differentiation grade. Furthermore, in patients with moderate/poorly differentiated OSCC, those with low Annexin A1 expression may potentially benefit from TPF induction chemotherapy on the aspect of OS and DMFS, especially DMFS. Patients with low Annexin A1 expression may benefit from TPF induction chemotherapy compared to those with high Annexin A1 expression.

Although the precise mechanism of Annexin A1 in cancer development and progression is still not clearly understood, more emphasis has been placed on this protein in the field of carcinogenesis, cancer diagnosis and cancer treatment. Annexin A1 has been previously linked with various cancers as a tumor suppressor protein. This includes breast cancer, head neck cancer, prostate cancer, cervical cancer, lung cancer [11-19]. However, increased Annexin A1 expression has also been reported in breast cancer, bladder cancer, pancreatic cancer, liver cancer, esophageal cancer, lung cancer [20-27]. Recently, the prognostic value of Annexin A1 expression has been reported in lung cancer, head neck cancer, bladder cancer and breast cancer [18-24], most of which report Annexin A1 overexpression indicates a poorer prognosis. In contrast, Annexin A1 overexpression in breast cancer correlates with a better survival [19]. In our study, a high Annexin A1 expression in the biopsy specimens indicated a poor prognosis, suggesting that Annexin A1 could be used as a prognostic biomarker for locally advanced OSCC. With respect to the role of Annexin A1 as a predictive biomarker for induction chemotherapy, future studies are necessary.

The correlation between Annexin A1 expression and pathologic differentiation grade has also been reported in several kinds of cancers, such as thyroid cancer, cervical cancer and head neck cancer [11-15,34]. In this study, we confirmed that the correlation between Annexin A1 expression and pathologic differentiation grade in OSCC. The proportion of patients with low Annexin A1 expression was higher amongst the patients with moderate/poorly differentiated tumor than those with well differentiated tumor. There was no significant difference between the pathologic differentiation grade and prognosis in both experimental and control groups. Radical removal of primary lesions as well as full neck dissection to eradicate as many lesions as possible may be an important factor for this result.

Correlation between Annexin A1 expression and response to induction chemotherapy has not been well documented. Absence of Annexin A1 expression coupled with presence of Annexin A2 expression is reported to correlate with a poor pathological response to induction chemotherapy in breast cancer [28]. Increased Annexin A1 expression is reported to correlate with anti-cancer drug resistance in some tumor cells in vitro [35]. In this study, we failed to find a significant correlation between Annexin A1 expression and response to TPF induction chemotherapy in OSCC. Moreover, Annexin A1 was found to have limited utility as a predictive marker of clinical or pathologic response to TPF induction chemotherapy when we looked at the entire cohort of patients that received induction chemotherapy. However, a subgroup analysis showed that in the patients with moderate/poorly differentiated tumor, low Annexin A1 expression did have an OS and DMFS benefit from TPF induction chemotherapy. This suggests that detection of Annexin A1 expression prior to treatment can be used to guide treatment selection. One can envision a personalized treatment scenario in which OSCC patients with moderate/poorly differentiated tumor and low Annexin A1 expression receive TPF induction chemotherapy prior to surgery while those with high Annexin A1 expression, receive surgery to avoid the toxicity from chemotherapeutic agents and the delay of definitive treatment. A limitation of our study is a small sample size of 78 patients with low Annexin A1 expression who were used for subgroup analysis. As such, these results need to be considered exploratory and hypothesis generating, and clearly need to be confirmed in further clinical trials with larger sample sizes.

In addition to our findings related to Annexin A1 expression, other biomarkers have been evaluated as potential predictors of benefit from TPF induction treatment in HNSCC patients. Higher beta-Tubulin-II and lower cyclin D1 have been found to strongly associate with lower response rates to TPF induction chemotherapy [36,37]. However, before being widely embraced, further clinical trials on prognostic and/or predictive biomarkers, alone or in combination, are needed to validate their clinical utility, and to realize the goal of personalized treatment for patients with HNSCC.

## Conclusions

Out studies suggest that Annexin A1 expression may serve as a prognostic biomarker in patients with resectable locally advanced OSCC. Furthermore, Annexin A1 is a predictive biomarker for response to TPF induction chemotherapy in patients with moderate/poorly differentiated OSCC.

## Competing interest

The authors declare that they have no competing interests.

## Authors’ contributions

LZ was responsible for the study design, interpretation of the data and revision of the manuscript. DZ and YL were responsible for data acquisition, analysis of the work presented and the preparation of the manuscript. X Yang, CY, JM, X Yang, JQ, LW, JL, CZ and ZZ participated in the clinical management of the patients. All authors read and approved the final manuscript.

## Pre-publication history

The pre-publication history for this paper can be accessed here:

http://www.biomedcentral.com/1471-2407/13/301/prepub
